# Answer to H. S's Case of Obstinate Gleet

**Published:** 1807-08

**Authors:** 


### Answer to H. S's Case of Obstinate Gleet.

Sir,

YOU observe, that in the first instance the disease was infectious; it must then have been gonorrhœa, and from the irritative fever induced I should suppose virulent. In men we often meet with cases, where virulent gonorrhœa terminates in gleet, or excretion of the mucous glands of the urethra; but in women this rarely happens, they have the disease at all times more mild, their urethra being shorter. They cannot be affected with swelled testicle, phymosis or paraphymosis; nor have they great chance of stricture in the urethra. But sometimes, when the affection is violent, they have very painful erections of the clitoris, accompanied with menorrhagia, or symptoms resembling it. I am apt to think, that the vagina is not unfrequently the seat of gonorrhœa as well as the urethra.

In

In this case, the mode of treatment employed by H. S. was very proper, and it is surprising that it was not attended with complete success. He observes, that the seat of the disease is evidently the urethra, " *as all the discharge proceeds entirely from it, and not from the vagina,* and that the eruption has never entirely left her. I am of opinion, that the pustular eruptions in this case, are occasioned by the discharge being of an acrid nature; they will sometimes proceed from stimulant applications, as ung. hydrag. fort. but, wherever they appear, there is always inflammation, as every pustule may be considered as a small abscess. A gleet is never known to produce either inflammation or pustular eruption, and the discharge is neither acrid nor infectious.

I would recommend to H. S. to discontinue giving medicines internally, except a decoction of the woods. To trust entirely to Dr. Kinglake's remedy for gout—cold water; frequently bathing with this, and drying the parts completely afterwards with a soft towel, and then sprinkling the pustular eruption with oxidum zinci præparatum, will very soon effect a cure. A. F

